# Parkinson's disease related pain: a review of recent findings

**DOI:** 10.1007/s00415-012-6754-5

**Published:** 2012-11-23

**Authors:** A. Truini, M. Frontoni, G. Cruccu

**Affiliations:** Department of Neurology and Psychiatry, University Sapienza, Viale Università 30, 00185 Rome, Italy

**Keywords:** Neuropathic pain, Central pain, Parkinson disease

## Abstract

In this review we summarize progress in research on Parkinson's disease-related pain as reported in articles published in the Journal of Neurology in the years 2011 and 2012.

## Introduction

As well as the well-known motor symptoms, patients with Parkinson's disease (PD) commonly experience pain [1]. Published studies indicate a prevalence ranging from 40 to 85 % (mean 67.6 %) [2]. Pain severely impairs parkinsonian patients’ quality of life and some patients find this non-motor symptom more distressing than motor disturbances.

Like motor symptoms, non-motor symptoms including pain arise through mechanisms involving nociceptive inputs to the basal ganglia [3]. Animal experiments show that morphine microinjected into the striatum and substantia nigra produces a naloxone-reversible analgesia unrelated to motor function [4]. In rats with experimentally-induced painful neuropathy striatal activity increased and opioids suppressed this increase [5]. Nociceptive inputs travel to the striatum from the various pain-related cortical areas including SII, insula, cingulate cortex, prefrontal areas, and thalamic intralaminar nuclei [6]. The nociceptive-responsive striatal neurons have large receptive fields and strongly respond as the noxious input increases in intensity, thus suggesting that rather than spatially localizing pain the striatum codes its nociceptive input intensity [7]. The basal ganglia also connect with several pain-related areas. Substantia nigra efferent pathways connect to amygdala, prefrontal cortex and cingulate cortex, areas involved in the motivational-affective pain dimension [8]. Neuroimaging studies in humans show that pain modulation involves striatal dopamine D2 receptors [9]. All these data suggest that abnormal basal ganglia function in PD modulates pain directly by increasing or diminishing nociceptive signal propagation, and indirectly by influencing affective and cognitive processes thereby regulating how patients expect, experience and interpret nociceptive signals and pain.

Patients with PD experience two different types of pain, nociceptive and neuropathic. Nociceptive pains are extremely frequent (accounting for 40–90 % of reported pain) [1]. Nociceptive pain related to PD is typically musculoskeletal and visceral. Musculoskeletal pain usually originates from abnormal posture, rigidity, and akinesia causing motor fluctuations, thus leading to painful dystonia. The most typical painful dystonia, reported by about 40 % of patients with PD, is that manifesting in the early morning, when dopaminergic stimulation is low and akinesia and rigidity are severe. Early morning dystonia manifests as a focal dystonia with plantar flexion and foot inversion. Another frequent type of nociceptive pain is the visceral pain that frequently accompanies constipation. Bowel functioning in patients with PD depends on several factors including autonomic failure involving the enteric nervous system. Dystonic contractions involving the anal sphincter can cause painful anismus.

PD-related neuropathic pain comprises radicular pain and central Parkinson's pain. Radicular pain has a higher prevalence in patients with PD than in the general population (14–35 % vs. 10 %) [1, 10]. This high frequency probably reflects the lumbar discal structure damage due to festination, kyphosis and dystonia. Pain directly related to PD (central Parkinson's pain) is a relatively rare condition (4–10 % of patients) [1, 11]. It mainly affects the body side with predominant motor symptoms and patients usually describe it as a burning, cramping sensation. Unlike “classic” central pain, central Parkinson's pain has no association with the clinically evident sensory deficits that reflect afferent pathway damage [12]. Although the data argues against the currently accepted criteria for defining neuropathic pain given that the diagnosis requires a sensory deficit, most investigators believe that this type of pain arises directly from basal ganglia dysfunction that alters sensory processing of nociceptive inputs [1, 11].

Over the past 2 years interest in problems related to pain in PD has enormously increased. This article reviews and summarizes the new findings in PD-related pain published in the Journal of Neurology in the years 2011 and 2012.

## Clinical information on pain related to PD

Virtually all patients with PD have non-motor symptoms, the most frequent being psychiatric symptoms and pain [13]. Non-motor symptoms may even, though rarely, precede the onset of the typical motor disorders. This pre-motor stage reflects the early structural changes taking place in the lower brainstem nuclei and peripheral nervous system, including the autonomic and enteric ganglia. Identifying the pre-motor stage could be clinically useful, because early treatment might influence the disease course, improving the long-term outcome. Pain is one of the most common pre-motor symptoms of PD, and can affect the patient’s life for years before PD is diagnosed. In their study highlighting the importance and high frequency of pain as a non-motor symptoms, Winkler and colleagues [14] proposed a PD risk score, grading various non-motor items including pain to define a risk profile for identifying patients with PD who might benefit from disease-modifying strategies. Whether managing PD early implies a better outcome also in non-motor symptoms, such as pain, is an open matter. To answer the question we need studies designed to assess the PD-related pain burden in relationship to disease-modifying drugs and treatment timing.

Although most studies report that PD-related pain is significantly more frequent in women than in men [11], some articles state the contrary [15]. Aiming at analysing the gender differences in non-motor PD symptoms, including pain, Martinez-Martin and colleagues studied for study 950 patients with PD [16]. Using the Non-Motor Symptom Score they investigated 30 items, including pain. Whereas no differences were found in age, age at onset, duration of disease, and motor disability between genders, pain was significantly more frequent in women than in men. This gender-related difference in pain in PD is in line with the many epidemiological studies showing that various acute and chronic pains, such as headache, oro-facial, musculoskeketal and abdominal pains are more frequent in women than in men. Although some sex-related differences in pain might reflect cognitive and social factors, the lower pain threshold and pain tolerance in women might arise partly from biological differences. Several experimental studies have documented gender differences in opioid analgesia suggesting that the endogenous pain inhibitory system is less efficient in women than in men [17]. The gender differences in PD-related pain, reported in the study by Martin and colleagues, could be used to improve pain management in PD for both genders, and analyze gender-related differences in outcome after similar treatments.

Although many studies show that PD-related pain adversely influences patients’ quality of life [18, 19], no study has investigated how pain affects daily activities. The study by Trail and colleagues [20], examining the relationships among activity and daily energy expenditure, non-motor symptoms and body mass index, surprisingly demonstrates that pain affects patients’ daily activities more than their memory problems and depression. Although this study admirably highlights the pain burden in daily activity in PD, unfortunately, because it was conducted in a veteran’s hospital, it almost exclusively enrolled men (98 %). Because pain is more frequent and severe in women than in men [21], this collection bias might weaken the study’s clinical significance on the impact of pain in patients with PD.

Managing PD and its related non-motor symptoms, including pain, exacts a considerable economic burden, especially given worldwide population aging. Intense research efforts, therefore, focus on reducing costs by developing new treatments and models for care. These developments oblige the national reimbursement agencies to undertake an accurate cost-effectiveness analysis. Although many studies have examined how non-motor symptoms and pain influence quality of life, little is known about health state utility values (the measure used in health economics for assessing the individual’s preferences for different health outcomes). In their study [22] in patients with a recently diagnosed PD, Shearer and colleagues have investigated health state utility values and estimated their clinical and demographic correlates for future economic studies. Besides providing data useful for cost-effectiveness analysis the investigators also report that pain can have a greater impact on quality of life than motor symptoms, thus underlining how important it is to manage non-motor symptoms more effectively in newly diagnosed PD.

Although the many studies addressing the problem of non-motor symptoms in PD have reported their high frequency and clinical importance, they used only short-term prospective observation periods. Hence, we know little about how these symptoms progress and how they contribute to deteriorating quality of life during the disease course. As the disease progresses, rigidity and akinesia producing musculoskeletal abnormalities and fluctuating motor symptoms causing painful dystonia (such as the early morning dystonia) increase [23]. Hence, the frequency and severity of pain should increase in parallel. The large prospective PRIAMO study (Parkinson's and non-motor symptoms) [24] collecting 707 patients and assessing symptoms related to 12 different non-motor domains over 24 months reports that as overall non-motor symptoms increase in number along with disease motor severity and duration. But whereas some domains such as sleep, gastrointestinal symptoms, attention/memory disturbances and skin disorders (hyperhidrosis and seborrhoea) become more prevalent, psychiatric, cardiovascular, and respiratory symptoms become less prevalent. Unexpectedly, the frequency and clinical importance of pain remained basically stable. The study reported that the number of patients complaining of pain increased by only 1 %, and the 39-item Parkinson's Disease Questionnaire (PDQ-39), the scale used for quality of life assessment [25] remained unchanged. Although this study provides the previously unavailable information on how pain influences disease progression and quality of life in PD it has two important limitations. It fails to assess symptom severity, or take medications into account. One reason why the study by Antonini et al. [23] found no significant differences in pain incidence might be that patients changed the pain-killers or antiparkinsonian medications they used during the study. Consistently, in most patients (more than 80 %) the Hoehn and Yahr scale remained unchanged during follow-up, thus suggesting stable motor control, probably because pharmacological treatment changed during the course of the disease. Further studies investigating pain progression should probably include data for medications used in their results.

## Pathophysiology

Whereas animal studies underline the role of the basal ganglia and dopaminergic transmission in pain, the mechanisms underlying pain related to PD in humans remain unclear. Currently only indirect evidence suggested nociceptive system dysfunction in patients with PD. The study by Zambito and colleagues [26] adds information on how PD can put patients at higher risk of pain than healthy subjects. Using electrical stimulation they assessed tactile threshold, pain threshold, and pain tolerance in 106 PD patients (66 of whom had chronic pain). They found that pain threshold and pain tolerance were significantly lower in PD patients than in control subjects, whereas tactile threshold values were comparable in both groups. Surprisingly pain threshold and pain tolerance tend to lower as PD progresses, thus suggesting that as basal ganglia dysfunction worsens, nociceptive system involvement increases. Unexpectedly no difference was found in pain thresholds and pain tolerance between PD patients with and without pain. This finding seems to argue against the possibility that decreased sensory thresholds play some role in the development of pain in PD patients, but when Zambito et al., analyzed sensory thresholds they failed to distinguish between neuropathic and nociceptive pain. This distinction is important because whereas nociceptive pain is presumably unrelated to changes in sensory thresholds, neuropathic pain, a pain type that directly involves the nociceptive pathways, is associated with abnormal sensory thresholds [27].

## Treatment options

The treatment of PD-related pain hinges on empirical data alone and no large randomized controlled data are available. The pharmacological management relies on antiparkinson, antinociceptive and antineuropathic medications. Deep brain stimulation (DBS) targeted to the subthalamic nucleus and globus pallidus, a surgical procedure widely used for treating motor disorders in PD [28, 29], effectively relieves various types of PD-related pain, such as musculoskeletal, dystonic and central Parkinson pain [30, 31]. Despite these successful DBS applications in their prospective study, Capelle and colleagues [32] showed that DBS targeted to the subthalamic nucleus and globus pallidus has only a mild and negligible effect on an unusual type of PD-related pain: the back pain due to camptocormia (the rare and disabling condition provoking abnormal trunk flexion). This finding implies that this condition, unlike segmental dystonia, arises through other still unknown mechanisms, insensitive to DBS. The study conducted by Capelle et al. provides useful information indirectly supporting the idea that PD-related pain is a heterogeneous condition arising through multiple and diverse mechanisms, and may help in defining a PD pain profile that responds to DBS [33].

## Conclusions and future directions

Studies dealing with PD-related pain published in the Journal of Neurology over the past 2 years highlighting the importance of pain in PD have provided new insights into this common problem. Despite advancing our knowledge in this field they leave many questions open. Most important, how should we categorize PD-related pain? Few studies reliably distinguish the various types of PD-related pain. Because the various types of pain arise from different mechanisms, and probably respond differently to treatment [34], the major need is to classify pain according to its pathophysiological mechanisms and use the information to plan reliable pharmacological trials.

Although no randomized controlled trials have yet investigated PD-related pain, some pharmacological and non-pharmacological trials are now underway (www.clinicaltrial.gov). We hope they will provide the information on treatment that can translate into clinically important advances for patients with PD.
